# *Staphylococcus aureus* and Staphylococcal Food-Borne Disease: An Ongoing Challenge in Public Health

**DOI:** 10.1155/2014/827965

**Published:** 2014-04-01

**Authors:** Jhalka Kadariya, Tara C. Smith, Dipendra Thapaliya

**Affiliations:** Department of Biostatistics, Environmental Health Sciences and Epidemiology, Kent State University, College of Public Health, 750 Hilltop Drive, Kent, OH 44242, USA

## Abstract

Staphylococcal food-borne disease (SFD) is one of the most common food-borne diseases worldwide resulting from the contamination of food by preformed *S. aureus* enterotoxins. It is one of the most common causes of reported food-borne diseases in the United States. Although several Staphylococcal enterotoxins (SEs) have been identified, SEA, a highly heat-stable SE, is the most common cause of SFD worldwide. Outbreak investigations have found that improper food handling practices in the retail industry account for the majority of SFD outbreaks. However, several studies have documented prevalence of *S. aureus* in many food products including raw retail meat indicating that consumers are at potential risk of *S. aureus* colonization and subsequent infection. Presence of pathogens in food products imposes potential hazard for consumers and causes grave economic loss and loss in human productivity via food-borne disease. Symptoms of SFD include nausea, vomiting, and abdominal cramps with or without diarrhea. Preventive measures include safe food handling and processing practice, maintaining cold chain, adequate cleaning and disinfection of equipment, prevention of cross-contamination in home and kitchen, and prevention of contamination from farm to fork. This paper provides a brief overview of SFD, contributing factors, risk that it imposes to the consumers, current research gaps, and preventive measures.

## 1. Introduction

Food-borne diseases are a major public health concern worldwide [1, 2]. WHO defines food-borne disease (FBD) as “disease of infectious or toxic nature caused by, or thought to be caused by, the consumption of food or water” [2]. Annually, an estimated 76 million illnesses, 325,000 hospitalizations, and 5,000 deaths are caused by food-borne diseases in the United States [3]. Among these cases, 31 known pathogens cause 9.4 million illnesses, 56,000 hospitalizations, and 1300 deaths [4]. Using data from 2000–2008, researchers estimated that pathogens that were implicated in most FBD were norovirus (5.5 million, 58%), nontyphoidal* Salmonella* spp. (1.0 million, 11%),* Clostrodium perfringens* (1.0 million, 10%), and* Campylobacter* spp. (0.8 million, 9%). Among many food-borne pathogens, nontyphoidal* Salmonella* spp. and* Campylobacter* spp. are the leading causes of FBD in the United States, England, and Australia [4].

*S. aureus* is a significant cause of FBD, causing an estimated 241,000 illnesses per year in the United States [4]. However, the true incidence of* Staphylococcus aureus* food-borne disease (SFD) could be a lot higher as sporadic food-borne disease caused by* S. aureus* is not reportable in the United States [5]. Some other contributing factors for the low incidence of SFD include misdiagnosis, improper sample collection and laboratory examination [6], lack of seeking medical attention by the affected persons complicating the laboratory confirmation [5, 7], and lack of routine surveillance of clinical stool specimens for* S. aureus* or its enterotoxins [5, 8, 9]. Unavailability of implicated foods for confirmation of laboratory testing at the time of outbreak investigation further complicates the matter [5]. It is essential to note that FBD that is confirmed by laboratory testing and reported to public health agencies accounts for only a small fraction of illnesses [4]. FBD impose a great economic burden, accounting for $50–$80 billion annually in “health care costs, lost productivity, and diminished quality of life” in the United States [10, 11]. It is estimated that each case of SFD costs $695, representing a total cost of $167,597,860 annually in the United States [10]. The Institute of Medicine recognized FBD as a high priority [12]. “The potential for foods to be involved in the emergence or reemergence of microbial threats to health is high, in large part because there are many points at which food safety can be compromised.” Although FBD has decreased in recent years, it is still higher than* Healthy People 2020* goals [10]. The presence of food-borne pathogens in ready-to-eat foods, meat, and meat products puts consumers at high risk and imposes grave economic losses to producers due to recalls of implicated food products [13, 14].

## 2. *Staphylococcus aureus*

*S. aureus* is a commensal and opportunistic pathogen that can cause wide spectrum of infections, from superficial skin infections to severe, and potentially fatal, invasive disease [15]. This ubiquitous bacterium is an important pathogen due to combination of “toxin-mediated virulence, invasiveness, and antibiotic resistance.” This organism has emerged as a major pathogen for both nosocomial and community-acquired infections.* S. aureus* does not form spores but can cause contamination of food products during food preparation and processing.* S. aureus* can grow in a wide range of temperatures (7° to 48.5° C; optimum 30 to 37°C), pH (4.2 to 9.3; optimum 7 to 7.5), and sodium chloride concentration up to 15% NaCl.* S. aureus* is a dessication tolerant organism with the ability to survive in potentially dry and stressful environments, such as the human nose and on skin and inanimate surfaces such as clothing and surfaces [16]. These characteristics favor growth of the organism in many food products [2].* S. aureus* can remain viable on hands and environmental surfaces for extended durations after initial contact [17, 18].

## 3. Staphylococcal Food-Borne Disease

SFD is one of the most common FBD and is of major concern in public health programs worldwide [1, 2, 19]. It is one of the most common causes of reported FBD in the United States [1, 20–22]. The first documented event of SFD due to the consumption of contaminated cheese was investigated by Vaughan and Sternberg in Michigan, USA, in 1884 [19]. A typical FBD caused by* S. aureus* has a rapid onset following ingestion of contaminated food (usually 3–5 hours). This is due to the production of one or more toxins by the bacteria during growth at permissive temperatures [2]. However, the incubation period of SFD depends on amount of toxin ingested [22]. Very small dose of SEs can cause SFD. For example, one report indicated that approximately 0.5 ng/mL concentration of SEs contaminated with chocolate milk caused a large outbreak [22, 23].

The onset of SFD is abrupt. Symptoms include hypersalivation, nausea, vomiting, and abdominal cramping with or without diarrhea. If significant fluid is lost, physical examination may reveal signs of dehydration and hypotension [1, 6, 22, 24]. Abdominal cramps, nausea, and vomiting are the most common [2]. Although SFD is generally self-limiting and resolves within 24–48 hours of onset, it can be severe, especially in infants, elderly, and immune-compromised patients [1, 6, 22]. Antibiotics are not used for therapy [7]. Approximately 10% of individuals inflicted with SFD will present to a hospital [22, 24]. Management of SFD is supportive. The attack rate of SFD can be up to 85% [22].* S. aureus* may not be detected by culture in the events when food is contaminated and toxin is formed prior to cooking [22, 25]. A study involving 7126 cases indicated that case fatality rate of SFD is 0.03%.; all deaths were in elderly patients [22]. Recovery is complete in approximately 20 hours [22, 24].

The conclusive diagnostic criteria of SFD are based upon the detection of staphylococcal enterotoxins in food [26], or recovery of at least 10^5^ *S. aureus *g^−1^ from food remnants [19].* S. aureus* enterotoxin can be detected on the basis of three types of methods: bioassays, molecular biology, and/or immunological techniques [19, 27]. Polymerase chain reaction (PCR), reverse transcription PCR (RT-PCR), and RT-quantitative PCR can be carried out to evaluate the toxic potential of strain [19]. The enzyme immunoassay and enzyme-linked fluorescent assay are the most commonly used immunological methods based on the use of antienterotoxin polyclonal or monoclonal antibodies [19]. Several molecular typing methods are widely used for the genetic characterization of* S. aureus* such as multilocus sequence typing,* spa* typing, SCC*mec* typing, and Pulse-field gel electrophoresis (PFGE). These techniques provide means to trace epidemiologically related strains leading to the tracking back to the origin of contamination [28]. However, these methods have variation in their discriminating powers and can be increased by combining the methods [29]. Molecular-based methods provide information about the source of contamination (human or animal origin). The PFGE and* spa* typing can be used alone or in association to gather the information regarding the origin of* S. aureus* contamination [19].

Various types of foods serve as an optimum growth medium for* S. aureus.* Foods that have been frequently implicated in SFD are meat and meat products, poultry and egg products, milk and dairy products, salads, bakery products, especially cream-filled pastries and cakes, and sandwich fillings [2, 6, 30]. Foods implicated with SFD vary from country to country, particularly due to variation in consumption and food habits [2]. If food is prepared in a central location and widely distributed, SFD outbreaks can have grave consequences impacting thousands of people. For example, over 13,000 cases of SFD occurred in Japan in 2000 as a result of contamination of milk at a dairy-food-production plant [22, 31].

## 4. *Staphylococcal aureus *Enterotoxins

*S. aureus* produces wide arrays of toxins. Staphylococcal enterotoxins (SEs) are a family of nine major serological types of heat stable enterotoxins (SEA, SEB, SEC, SED, SEE, SEG, SEH, SEI, and SEJ) that belong to the large family of pyrogenic toxin superantigens [1, 6]. Pyrogenic toxins cause superantigenic activity such as immunosuppression and nonspecific T-cell proliferation [2]. It is hypothesized that superantigenic activity of SEs helps facilitate transcitosis that allows the toxin to enter the bloodstream, thus enabling it to interact with antigen-presenting cells and T cells leading to superantigen activity [1, 6, 19]. The majority of effects of SEs in SFD is believed to be triggered by initiating a focal intestinal inflammatory response due to their superantigenic activity or by affecting intestinal mast cells causing their degranulation [1, 22, 32].

SEs are highly stable and highly heat-resistant and resistant to environmental conditions such as freezing and drying [2, 19]. They are also resistant to proteolytic enzymes such as pepsin or trypsin and low pH, enabling them to be fully functional in the gastrointestinal tract after ingestion [2, 6]. The heat stability characteristic of* S. aureus* imposes a significant threat in food industries [1]. The mechanisms of SEs causing food poisoning are not clearly known. However, it is believed that SEs directly affect intestinal epithelium and vagus nerve causing stimulation of the emetic center [2, 19]. All staphylococcal enterotoxins cause emesis [22, 32]. An estimated 0.1 *μ*g of SEs can cause staphylococcal food poisoning in humans [2].

SEs produced by some strains of* S. aureus* are the causative agents of SFD, and SEA is the most common toxin implicated in such events. SEA is highly resistant to proteolytic enzymes. SEA was recovered from 77.8% of all SFD outbreaks in the United States followed by SED (37.5%) and SEB (10%) [1, 6]. SEA is the most commonly found enterotoxin among SFD outbreaks in Japan, France, and UK [6]. However, SEC and SEE are also implicated with SFD. The outbreak of gastrointestinal illness via contaminated coleslaw in the United States was caused by SEC produced by methicillin-resistant* S. aureus* (MRSA) from an asymptomatic food handler [33]. SEC was linked to the SFD outbreak in 1980 in Canada [34]. SEC was also involved in the SFD outbreak during 2001–2003 in Taiwan [35] and 2009 outbreak in Japan [36].* S. aureus* is often implicated with caprine mastitis [37]. In sheep, goats, and cattle, SEC was the predominant toxin type detected in* S. aureus* isolated from mastitis milk [38]. Other studies have documented SEC producers as the most prevalent enterotoxin-producing* S. aureus* isolated from goat's milk [39] and goat's skin of udder, teats, and milk [40]. Six SFD outbreaks in France in 2009 were caused by SEE present in soft cheese made from unpasteurized milk [26]. Although rare, SEE has also been implicated in the SFD outbreaks in USA and UK [6]. Various new SEs (SEG to SE*l*U2) have been identified. However, only SEH-producing strains have been involved in SFD outbreaks [6].

*S. aureus* can survive in multiple host species. Molecular typing such as multilocus sequence typing (MLST) has helped to gain insights about population structure of* S. aureus*. Studies have identified over 2200 sequence types (STs) of* S. aureus* using the MLST techniques. The STs can be grouped into clonal complexes (CC). Several studies have indicated that majority of the livestock-associated STs belong to a small number of animal-associated clones. For example, CC97, ST151, CC130, and CC126 are commonly found on bovine infections. CC133 are common among small-ruminants such as sheep or goats. ST1, ST8, CC5, ST 121, and ST398 are found in human host species [41]. ST5 is predominant among poultry isolates [42]. CC133 and ST522 are mostly implicated with mastitis in sheep and goats. One Danish study indicated that ST133 was the predominant lineage in sheep and goats [42].

## 5. Contributing Factors

In the United States, approximately 30% and 1.5% of the population are colonized with methicillin-susceptible* S. aureus* (MSSA) [43] and MRSA, respectively, [43–45] with the most important site for colonization being the anterior nares (nostrils) [46]. While colonization itself does not harm the host, it is a risk factor for developing subsequent symptomatic infections [43, 47]. These colonized healthy persons categorized as persistent carriage and intermittent carriage serve as* S. aureus* careers and are able to transmit the bacterium to susceptible persons [46].

*S. aureus* is a common causative agent of bovine mastitis in dairy herds. A study conducted in Minnesota to estimate the heard prevalence of* S. aureus* from bulk tank milk found that heard prevalence of MSSA and MRSA was 84% and 4%, respectively [48]. Other studies estimated that the prevalence of* S. aureus* in bulk milk tank was 31% in Pennsylvania and 35% in cow milk samples in Louisiana [48]. Studies from Argentina [49], Brazil [50], Ireland [51], and Turkey [52] have documented the presence of staphylococcal enterotoxin genes and production of SEs by* S. aureus* of bovine origin. The udders with clinical and subclinical staphylococcal mastitis can contribute to the contamination of milk by* S. aureus* via direct excretion of the organisms in the milk [38] with large fluctuations in counts ranging from zero to 10^8^ CFU/mL [53]. For example, cattle mastitis was the sole source of contamination in 1999* S. aureus* outbreak in Brazil that affected 328 individuals who consumed unpasteurized milk [54]. Similarly, 293* S*.* aureus* isolates were recovered from 127 bulk tank milk samples of goats and sheep from Switzerland [38]. Recently,* S. aureus* isolates were recovered from mammary quarter milk of mastitic cows and from bulk tank milk produced on Hungarian dairy farms indicating that* S. aureus* from infected udders may contaminate bulk milk and, subsequently, raw milk products [53]. However,* S. aureus* contamination in milk can occur from the environment during handling and processing of raw milk as well [53].

Improper food handling practices in the retail food industry are thought to contribute to a high number of FBD outbreaks [55]. Studies have indicated that the majority of FBD outbreaks result from such practices [55, 56]. It was reported that the hands of food handlers were implicated in 42% of food-borne outbreaks that occurred between 1975 and 1998 in the United States [55, 57].

In a recent study [13] investigating the microbiological contamination in ready-to-eat food products processed at a large processing plant in Trinidad, West Indies,* S. aureus* was the most common pathogen detected.* S. aureus* was isolated from precooked food samples of franks, bologna, and bacon and postcooked bologna and bacon. The overall prevalence of* S. aureus* detected in air, food, and environmental samples was 27.1% (46/170). It was determined that the counts of* S. aureus* increased after heat treatment, and only postcooking environmental surfaces that came into contact with ready-to-eat foods that were contaminated with* S. aureus* during slicing and packaging harbored* S. aureus*.* S. aureus* was also frequently found on food handler's gloves [13]. Pathogenic microbes can adhere to the surface of the gloves worn by retail food employees and can serve as a source of cross-contamination if not changed frequently [55]. The practice of wearing gloves without proper hand washing can contaminate both the interior and exterior of the gloves. Hand washing is often neglected when gloves are used, which may promote rapid microbial growth on the hands as gloves provide a warm, moist environment for bacterial growth on the hands [55, 57]. Hand-washing, an easy method of preventing many microbial contamination, is too often forgotten [55].

The finding of high bacterial counts in the air and on food contact surfaces in the postprocessing environment is suggestive of cross-contamination of postcooked products and is the most important risk factor affecting microbiological quality of food [13]. A study [58] found that processed foods that require more handling during preparation are more vulnerable to* S. aureus* contamination [13]. Another study [59] demonstrated that increased human handling contributed to contamination by* S. aureus* in a pork processing plant.

Analysis of the data of FBD outbreaks reported to the Food-borne Disease Outbreak Surveillance System during 1998 to 2008 [5] indicate that meat and poultry dishes were the most common foods (55% of* S*.* aureus* outbreaks) reported in* S. aureus* outbreaks in the United States. Foods implicated with* S. aureus* outbreaks were most often prepared in a restaurant or deli (44%). Errors in food processing and preparation (93%) were the most common contributing factor in FBD outbreaks. Forty-five percent and 16% of these errors occurred in restaurants and delis and homes or private residences, respectively. The study identified various errors in food processing and preparation that include (i) insufficient time and temperature during initial cooking (40%) hot holding (33%) and reheating process (57%); (ii) prolonged exposure of foods at room or outdoor temperature (58%); (iii) slow cooling of prepared food (44%); (iv) inadequate cold holding temperatures (22%); (v) and preparing foods for extended periods of time prior to serving [5]. Cross-contamination in the vicinity of food preparation and processing was another contributing factor in* S. aureus* food-borne outbreaks. Insufficient cleaning of processing equipment or utensils (67%) and storage in contaminated environments (39%) were the most common errors reported [5].

## 6. Farm, Food, and Beyond

In recent years, a new strain of* S. aureus*, livestock-associated methicillin-resistant* Staphylococcus aureus* (LA-MRSA), has been recognized as a novel pathogen that has become a rapidly emerging cause of human infections [60, 61]. LA-MRSA was first detected in 2005 in swine farmers and swine in France and in The Netherlands [62–64]. Researchers have isolated LA-MRSA from number of countries in Asia [65–67], Europe [68–74], and North America [75, 76]. Studies have found increased human colonization and infection of LA-MRSA belonging to the multilocus sequence type 398 (ST398) lineages in livestock-dense areas in Europe [77–80]. Investigators in The Netherlands have shown that ST398 now accounts for 20% of human MRSA cases [81] and this strain accounts for 42% of newly detected MRSA in that country, suggesting that animals may be an important reservoir for human MRSA infections [77]. Compared to the general population, Dutch pig farmers are 760 times more likely to be colonized with MRSA [82].

In several studies, MRSA has been found at high levels on US and European farms and in commercially-distributed meats, emerging as a potential concern for meat handlers and consumers [28, 64, 68, 75–77, 83–88]. Several species of meat-producing animals are frequently implicated including pigs [68, 75, 76], poultry [89–91], and cattle [73, 92]. The presence of MRSA on raw retail meat products is well documented, with prevalence ranging from less than 1 percent in Asia [93, 94] to 11.9% in The Netherlands [95], with intermediate prevalence found in other studies [87, 96, 97]. A recent study carried out in the United States found that 45% (45/100) of pork products and 63% (63/100) of beef products tested in Georgia were positive for* S. aureus*. The MRSA prevalence in this study was 3% and 4% in retail pork and beef, respectively [28]. Another US study testing retail meat in Louisiana isolated MRSA from 5% (6/120) of meat samples tested, while 39.2% (47/120) of samples were positive for any type of* S. aureus* [87]. Very high prevalence of* S. aureus* (64.8%, 256/395) was observed on retail pork products collected from Iowa, Minnesota, and New Jersey [85]. The prevalence of MRSA in this study was 6.6%. Other studies in US have found* S. aureus* in 16.4% (27/165) and MRSA in 1.2% (2/165) of meat samples [84], multidrug resistant (MDR)* S. aureus* in 52% (71/136) of meat and poultry samples [86], and any* S. aureus* in 22.5% (65/289) and MRSA in 2% (6/289) of meat and poultry samples [88]. These studies provide some insights regarding the role of commercially distributed meat as a potential vehicle for* S. aureus* transmission from the farm into the general human population.

The first report of an outbreak of gastrointestinal illness caused by a community-acquired methicillin resistant* S. aureus* in the United States affected 3 members of the same family. Contaminated coleslaw from an asymptomatic food handler was the source of MRSA [6, 33]. All 3 members of the family who ate foods (shredded pork barbeque and coleslaw) 30 minutes after purchasing at a convenient-market delicatessen developed gastrointestinal symptoms. The* S. aureus* isolates recovered from the stool samples of the three ill family members and coleslaw and nasal swab of food preparer were identical in PFGE analysis. The implicated strain produced Staphylococcal toxin C and was identified as MRSA [33].

This outbreak provides an evidence of MRSA-contaminated foods as the vehicle in the clusters of illness affecting low-risk persons within the community. The food handlers involved in this outbreak had visited a nursing home. It is important to note that many* S. aureus* isolates obtained as a part of outbreak investigation may not be tested for antibiotic susceptibility, as antibiotics are not used in the treatment regimen. As such, it is plausible that food-borne outbreak caused by methicillin-resistant strains of* S. aureus* may go unnoticed. Previously food has been implicated as a source of MRSA transmission in one outbreak of blood and wound infections in hospitalized immunocompromised patients [33, 98].

## 7. Gaps in Research

Many outbreak investigations successfully traced food handlers as a source of contamination matching the strains of* S. aureus* in food products and handlers. However, these retrospectively carried-out studies have some limitations and cannot ascertain that the handler was not also colonized due to the exposure to* S. aureus *contaminated food.

Although numerous studies have focused on documenting risk imposed by* S. aureus* toxins in food industry and consumers' health, little is known about the potential role of intact bacteria transmitted through the raw meat products and self-inoculation into the nasal cavity of food industry workers and consumers. Additionally, while research has shown the potential for transmission of* S. aureus* within the home setting [99, 100], the relationship of colonization and transmission of this organism to the food products brought into the home has not been investigated.

Several European studies investigating MRSA in retail meat found ST398 as the most common MRSA type [95, 97, 101]. It has been suggested that meat might be a potential vehicle for the transmission of ST398 from the farm into the community, but additional research needs to be carried out to test this hypothesis.

Researchers have isolated other non-ST398 strains of* S. aureus* such as ST8, a strain which includes USA 300, the primary cause of community-associated MRSA infections, from US swine farms [102] and retail meat [28, 84–88]. However, it is not clear whether human handlers played any role during the postslaughter processing for the contamination of meat positive for ST8. It is suggested that since* S. aureus* is also present in intestinal tract [103], raw meat may contain MRSA due to the carcasses contaminated with intestinal content during slaughtering process [95]. Finding of human-associated strains of MRSA from raw chicken meat in Japan and Korea provides some support to this hypothesis [89, 93, 94].

Only few studies have been conducted specifically to investigate the implication of MRSA in SFD [19]. Although MRSA was frequently isolated from food production animals and raw retail meat, the relevance of its contamination is unknown. Further study is warranted to investigate the likelihood of gastrointestinal colonization and extraintestinal infection subsequent to the consumption of foods contaminated with MRSA [104]. Since* S. aureus* isolates obtained from SFD outbreaks may not be tested for antibiotic susceptibility, the true prevalence of MRSA involved in SFD is unknown [33]. Since other Staphylococcal species are also able to produce SEs and are not routinely tested, further research is warranted [2].

## 8. Prevention

SFD is preventable [10]. Consumers need to be aware of potential food contamination in home and during cooking in kitchen. Cooking food thoroughly is important, but preventing contamination and cross-contamination and maintaining critical points are the most effective ways to prevent SFD. Since research findings and outbreak investigations have suggested that SFD is largely due to faulty food handling practices, knowledge and skills in food industry workers are warranted. Nevertheless, public health intervention should be designed to prevent* S. aureus* from pre- and postslaughter in meat processing facilities. Public awareness regarding safe meat handling would help to prevent cross-contamination [104] as well as potential colonization of handlers from contaminated food products. Other public health interventions such as personalized and tailored food safety education program targeting diverse sociodemographic people could be a cornerstone in preventing the SFD outbreak [10].

1985's staphylococcal food poisoning due to contaminated chocolate milk in Kentucky, USA, and 2000's extensive outbreak of staphylococcal food poisoning due to contaminated low-fat milk in Japan, are the classical examples of SFD that illustrate the stability and heat resistance of SEs as well as the importance of illumination of any contamination sources during the processing and refrigeration of food and food ingredients. In both cases, high temperature used in pasteurization killed the bacteria but had no effect on SEs [2, 31].

The permissive temperature for the growth and toxin production by* S. aureus* is between 6°C and 46°C. Thus, the ideal cooking and refrigerating temperature should be above 60°C and below 5°C, respectively. A study reviewing the performance of domestic refrigerators worldwide found that many refrigerators were running above the recommended temperature [105]. Another study conducted in Portugal found that more than 80% of participants cleaned their fridge only monthly [106]. While these studies indicate the need of consumer awareness in food safety, other preventive measures such as the practice of serving food rapidly when kept at room temperature, wearing gloves, masks, hairnets during food handling and processing, frequent hand washing, good personal hygiene of food handlers, and use of “sneeze-bars” at buffet tables could help prevent SFD [22, 58].

Maintaining the cold chain is essential for preventing the growth of* S. aureus* in food products [5]. Other preventive measures such as control of raw ingredients, proper handling and processing, adequate cleaning, and disinfection of equipment used in food processing and preparation should be deployed [19, 104]. Strict implementation and adherence to the microbiological guidelines such as Hazard Analysis and Critical Control Points (HACCP), Good Manufacturing Practice (GMPs), and Good Hygienic Practices (GHPs) developed by World Health Organization and United States Food and Drug Administration can help to prevent* S. aureus* contamination [13, 107].

## 9. Conclusion

SFD is one of the most common causes of FBD worldwide. Outbreak investigations have suggested that improper handling of cooked or processed food is the main source of contamination. Lack of maintaining cold chain allows* S. aureus* to form SEs. Although* S. aureus* can be eliminated by heat treatment and by competition with other flora in pasteurized and fermented foods, respectively, SEs produced by* S. aureus* are still capable of causing SFD because of their heat tolerance capacity. This fact should be considered in risk assessment and devising appropriate public health interventions. Prevention of* S. aureus* contamination from farm to fork is crucial. Rapid surveillance in the event of SFD outbreak and ongoing surveillance for the routine investigation of* S. aureus* and SEs implicated in food products along with improved diagnostic methods could help to combat the SFD in 21st century. Recent findings of high prevalence of* S. aureus* including MRSA in raw retail meat impose a potential hazard to consumers, both as classic SFD and as a potential source of colonization of food handlers. Further study is required to fill the research gap.

## References

[1] Balaban N, Rasooly A (2000). Staphylococcal enterotoxins. *International Journal of Food Microbiology*.

[2] le Loir Y, Baron F, Gautier M (2003). *Staphylococcus aureus* and food poisoning. *Genetics and Molecular Research*.

[3] Mead PS, Slutsker L, Dietz V (1999). Food-related illness and death in the United States. *Emerging Infectious Diseases*.

[4] Scallan E, Hoekstra RM, Angulo FJ (2011). Foodborne illness acquired in the United States—major pathogens. *Emerging Infectious Diseases*.

[5] Bennett SD, Walsh KA, Gould LH (2013). Foodborne disease outbreaks caused by Bacillus cereus, Clostridium perfringens, and *Staphylococcus aureus*—United States, 1998–2008. *Clinical Infectious Diseases*.

[6] Argudín MÁ, Mendoza MC, Rodicio MR (2010). Food poisoning and *Staphylococcus aureus* enterotoxins. *Toxins*.

[7] Scallan E, Jones TF, Cronquist A (2006). Factors associated with seeking medical care and submitting a stool sample in estimating the burden of foodborne illness. *Foodborne Pathogens and Disease*.

[8] Guerrant RL, van Gilder T, Steiner TS (2001). Practice guidelines for the management of infectious diarrhea. *Clinical Infectious Diseases*.

[9] Thielman NM, Guerrant RL (2004). Acute Infectious Diarrhea. *The New England Journal of Medicine*.

[10] Byrd-Bredbenner C, Berning J, Martin-Biggers J, Quick V (2013). Food safety in home kitchens: a synthesis of the literature. *International Journal of Environmental Research and Public Health*.

[11] Scharff RL (2012). Economic burden from health losses due to foodborne illness in the united states. *Journal of Food Protection*.

[12] Cohen ML (2000). Changing patterns of infectious disease. *Nature*.

[13] Syne SM, Ramsubhag A, Adesiyun AA (2013). Microbiological hazard analysis of ready-to-eat meats processed at a food plant in Trinidad, West Indies. *Infection Ecology & Epidemiology*.

[14] Sofos JN (2008). Challenges to meat safety in the 21st century. *Meat Science*.

[15] Lowy FD (1998). Medical progress: *Staphylococcus aureus* infections. *The New England Journal of Medicine*.

[16] Chaibenjawong P, Foster SJ (2011). Desiccation tolerance in *Staphylococcus aureus*. *Archives of Microbiology*.

[17] Kusumaningrum HD, van Putten MM, Rombouts FM, Beumer RR (2002). Effects of antibacterial dishwashing liquid on foodborne pathogens and competitive microorganisms in kitchen sponges. *Journal of Food Protection*.

[18] Scott E, Bloomfield SF (1990). The survival and transfer of microbial contamination via cloths, hands and utensils. *Journal of Applied Bacteriology*.

[19] Hennekinne J-A, de Buyser M-L, Dragacci S (2012). *Staphylococcus aureus* and its food poisoning toxins: characterization and outbreak investigation. *FEMS Microbiology Reviews*.

[20] Bean NH, Griffin PM, Goulding JS, Ivey CB (1990). Foodborne disease outbreaks, 5-year summary, 1983–1987. *CDC Surveillance Summaries*.

[21] Bunning VK, Lindsay JA, Archer DL (1997). Chronic health effects of microbial foodborne disease. *World Health Statistics Quarterly*.

[22] Murray RJ (2005). Recognition and management of *Staphylococcus aureus* toxin-mediated disease. *Internal Medicine Journal*.

[23] Evenson ML, Hinds MW, Bernstein RS, Bergdoll MS (1988). Estimation of human dose of staphylococcal enterotoxin A from a large outbreak of staphylococcal food poisoning involving chocolate milk. *International Journal of Food Microbiology*.

[24] Holmberg SD, Blake PA (1984). Staphylococcal food poisoning in the United States. New facts and old misconceptions. *Journal of the American Medical Association*.

[25] Eisenberg MS, Gaarslev K, Brown W, Horwitz M, Hill D (1975). Staphylococcal food poisoning aboard a commercial aircraft. *The Lancet*.

[26] Ostyn A, de Buyser ML, Guillier F (2010). First evidence of a food poisoning outbreak due to staphylococcal enterotoxin type E, France, 2009. *Euro Surveillance*.

[27] Mclauchlin J, Narayanan GL, Mithani V, O’Neill G (2000). The detection of enterotoxins and toxic shock syndrome toxin genes in *Staphylococcus aureus* by polymerase chain reaction. *Journal of Food Protection*.

[28] Jackson CR, Davis JA, Barrett JB (2013). Prevalence and characterization of methicillin-resistant *Staphylococcus aureus* isolates from retail meat and humans in Georgia. *Journal of Clinical Microbiology*.

[29] Wendlandt S, Schwarz S, Silley P (2013). Methicillin-resistant *Staphylococcus aureus*: a food-borne pathogen?. *Annual Review of Food Science and Technology*.

[30] Tamarapu S, McKillip JL, Drake M (2001). Development of a multiplex polymerase chain reaction assay for detection and differentiation of *Staphylococcus aureus* in dairy products. *Journal of Food Protection*.

[31] Asao T, Kumeda Y, Kawai T (2003). An extensive outbreak of staphylococcal food poisoning due to low-fat milk in Japan: estimation of enterotoxin A in the incriminated milk and powdered skim milk. *Epidemiology and Infection*.

[32] Dinges MM, Orwin PM, Schlievert PM (2000). Exotoxins of *Staphylococcus aureus*. *Clinical Microbiology Reviews*.

[33] Jones TF, Kellum ME, Porter SS, Bell M, Schaffner W (2002). An outbreak of community-acquired foodborne illness caused by methicillin-resistant *Staphylococcus aureus*. *Emerging Infectious Diseases*.

[34] de Buyser M-L, Dufour B, Maire M, Lafarge V (2001). Implication of milk and milk products in food-borne diseases in France and in different industrialised countries. *International Journal of Food Microbiology*.

[35] Chiang Y-C, Liao W-W, Fan C-M, Pai W-Y, Chiou C-S, Tsen H-Y (2008). PCR detection of Staphylococcal enterotoxins (SEs) N, O, P, Q, R, U, and survey of SE types in *Staphylococcus aureus* isolates from food-poisoning cases in Taiwan. *International Journal of Food Microbiology*.

[36] Kitamoto M, Kito K, Niimi Y (2009). Food poisoning by *Staphylococcus aureus* at a University festival. *Japanese Journal of Infectious Diseases*.

[37] de Buyser ML, Dilasser F, Hummel R, Bergdoll MS (1987). Enterotoxin and toxic shock syndrome toxin-1 production by staphylococci isolated from goat’s milk. *International Journal of Food Microbiology*.

[38] Scherrer D, Corti S, Muehlherr JE, Zweifel C, Stephan R (2004). Phenotypic and genotypic characteristics of *Staphylococcus aureus* isolates from raw bulk-tank milk samples of goats and sheep. *Veterinary Microbiology*.

[39] Foschino R, Invernizzi A, Barucco R, Stradiotto K (2002). Microbial composition, including the incidence of pathogens, of goat milk from the Bergamo region of Italy during a lactation year. *Journal of Dairy Research*.

[40] Valle J, Gomez-Lucia E, Piriz S, Goyache J, Orden JA, Vadillo S (1990). Enterotoxin production by staphylococci isolated from healthy goats. *Applied and Environmental Microbiology*.

[41] Fitzgerald JR (2012). Livestock-associated *Staphylococcus aureus*: origin, evolution and public health threat. *Trends in Microbiology*.

[42] Eriksson J, Espinosa-Gongora C, Stamphoj I, Larsen AR, Guardabassi L (2013). Carriage frequency, diversity and methicillin resistance of *Staphylococcus aureus* in Danish small ruminants. *Veterinary Microbiology*.

[43] Graham PL, Lin SX, Larson EL (2006). A U.S. population-based survey of *Staphylococcus aureus* colonization. *Annals of Internal Medicine*.

[44] Gorwitz RJ, Kruszon-Moran D, McAllister SK (2008). Changes in the prevalence of nasal colonization with *Staphylococcus aureus* in the United States, 2001–2004. *Journal of Infectious Diseases*.

[45] Smith TC, Gebreyes WA, Abley MJ (2013). Methicillin-resistant *Staphylococcus aureus* in pigs and farm workers on conventional and antibiotic-free swine farms in the USA. *PLoS ONE*.

[46] Wertheim HFL, Melles DC, Vos MC (2005). The role of nasal carriage in *Staphylococcus aureus* infections. *Lancet Infectious Diseases*.

[47] Fritz SA, Epplin EK, Garbutt J, Storch GA (2009). Skin infection in children colonized with community-associated methicillin-resistant *Staphylococcus aureus*. *Journal of Infection*.

[48] Haran KP, Godden SM, Boxrud D, Jawahir S, Bender JB, Sreevatsan S (2012). Prevalence and characterization of *Staphylococcus aureus*, including methicillin-resistant *Staphylococcus aureus*, isolated from bulk tank milk from Minnesota dairy farms. *Journal of Clinical Microbiology*.

[49] Neder VE, Canavesio VR, Calvinho LF (2011). Presence of enterotoxigenic *Staphylococcus aureus* in bulk tank milk from Argentine dairy farms. *Revista Argentina de Microbiologia*.

[50] Arcuri EF, Ângelo FF, Martins Guimarã Es MF (2010). Toxigenic status of *Staphylococcus aureus* isolated from bovine raw milk and Minas frescal cheese in Brazil. *Journal of Food Protection*.

[51] Murphy BP, O’Mahony E, Buckley JF, O’Brien S, Fanning S (2010). Characterization of *Staphylococcus aureus* isolated from dairy animals in ireland. *Zoonoses and Public Health*.

[52] Günaydin B, Aslantaş Ö, Demir C (2011). Detection of superantigenic toxin genes in *Staphylococcus aureus* strains from subclinical bovine mastitis. *Tropical Animal Health and Production*.

[53] Peles F, Wagner M, Varga L (2007). Characterization of *Staphylococcus aureus* strains isolated from bovine milk in Hungary. *International Journal of Food Microbiology*.

[54] Simeão do Carmo L, Dias RS, Linardi VR (2002). Food poisoning due to enterotoxigenic strains of Staphylococcus present in Minas cheese and raw milk in Brazil. *Food Microbiology*.

[55] Lues JFR, van Tonder I (2007). The occurrence of indicator bacteria on hands and aprons of food handlers in the delicatessen sections of a retail group. *Food Control*.

[56] Clayton DA, Griffith CJ, Price P, Peters AC (2002). Food handlers’ beliefs and self-reported practices. *International Journal of Environmental Health Research*.

[57] Ayçiçek H, Aydoğan H, Küçükkaraaslan A, Baysallar M, Başustaoğlu AC (2004). Assessment of the bacterial contamination on hands of hospital food handlers. *Food Control*.

[58] Aycicek H, Cakiroglu S, Stevenson TH (2005). Incidence of *Staphylococcus aureus* in ready-to-eat meals from military cafeterias in Ankara, Turkey. *Food Control*.

[59] Saide-Albornoz JJ, Lynn Knipe C, Murano EA, Beran GW (1995). Contamination of pork carcasses during slaughter, fabrication, and chilled storage. *Journal of Food Protection*.

[60] Larson KRL, Harper AL, Hanson BM (2011). Methicillin-resistant *Staphylococcus aureus* in pork production shower facilities. *Applied and Environmental Microbiology*.

[61] Price LB, Stegger M, Hasman H (2012). *Staphylococcus aureus* CC398: host adaptation and emergence of methicillin resistance in livestock. *MBio*.

[62] Smith TC, Pearson N (2011). The emergence of *Staphylococcus aureus* ST398. *Vector-Borne and Zoonotic Diseases*.

[63] Wulf M, Voss A (2008). MRSA in livestock animals—an epidemic waiting to happen?. *Clinical Microbiology and Infection*.

[64] Armand-Lefevre L, Ruimy R, Andremont A (2005). Clonal comparison of Staphylococcus from healthy pig farmers, human controls, and pigs. *Emerging Infectious Diseases*.

[65] Guardabassi L, O’Donoghue M, Moodley A, Ho J, Boost M (2009). Novel lineage of methicillin-resistant *Staphylococcus aureus*, Hong Kong. *Emerging Infectious Diseases*.

[66] Wagenaar JA, Yue H, Pritchard J (2009). Unexpected sequence types in livestock associated methicillin-resistant *Staphylococcus aureus* (MRSA): MRSA ST9 and a single locus variant of ST9 in pig farming in China. *Veterinary Microbiology*.

[67] Sergio DMB, Tse HK, Hsu L-Y, Ogden BE, Goh ALH, Chow PKH (2007). Investigation of meticillin-resistant *Staphylococcus aureus* in pigs used for research. *Journal of Medical Microbiology*.

[68] de Neeling AJ, van den Broek MJM, Spalburg EC (2007). High prevalence of methicillin resistant *Staphylococcus aureus* in pigs. *Veterinary Microbiology*.

[69] Lewis HC, Mølbak K, Reese C (2008). Pigs as source of methicillin-resistant *Staphylococcus aureus* CC398 infections in humans, Denmark. *Emerging Infectious Diseases*.

[70] Witte W, Strommenger B, Stanek C, Cuny C (2007). Methicillin-resistant *Staphylococcus aureus* ST398 in humans and animals, central Europe. *Emerging Infectious Diseases*.

[71] Guardabassi L, Stegger M, Skov R (2007). Retrospective detection of methicillin resistant and susceptible *Staphylococcus aureus* ST398 in Danish slaughter pigs. *Veterinary Microbiology*.

[72] Denis O, Suetens C, Hallin M (2009). Methicillin-resistant *Staphylococcus aureus* ST398 in swine farm personnel, Belgium. *Emerging Infectious Diseases*.

[73] Pomba C, Hasman H, Cavaco LM, da Fonseca JD, Aarestrup FM (2009). First description of meticillin-resistant *Staphylococcus aureus* (MRSA) CC30 and CC398 from swine in Portugal. *International Journal of Antimicrobial Agents*.

[74] Battisti A, Franco A, Merialdi G (2010). Heterogeneity among methicillin-resistant *Staphylococcus aureus* from Italian pig finishing holdings. *Veterinary Microbiology*.

[75] Smith TC, Male MJ, Harper AL (2009). Methicillin-resistant *Staphylococcus aureus* (MRSA) strain ST398 is present in midwestern U.S. swine and swine workers. *PLoS ONE*.

[76] Khanna T, Friendship R, Dewey C, Weese JS (2008). Methicillin resistant *Staphylococcus aureus* colonization in pigs and pig farmers. *Veterinary Microbiology*.

[77] Monaco M, Pedroni P, Sanchini A (2013). Livestock-associated methicillin-resistant *Staphylococcus aureus* responsible for human colonization and infection in an area of Italy with high density of pig farming. *BMC Infectious Diseases*.

[78] Köck R, Harlizius J, Bressan N (2009). Prevalence and molecular characteristics of methicillin-resistant *Staphylococcus aureus* (MRSA) among pigs on German farms and import of livestock-related MRSA into hospitals. *European Journal of Clinical Microbiology and Infectious Diseases*.

[79] Köck R, Siam K, Al-Malat S (2011). Characteristics of hospital patients colonized with livestock-associated meticillin-resistant *Staphylococcus aureus* (MRSA) CC398 versus other MRSA clones. *Journal of Hospital Infection*.

[80] Wulf MWH, Verduin CM, van Nes A, Huijsdens X, Voss A (2012). Infection and colonization with methicillin resistant *Staphylococcus aureus* ST398 versus other MRSA in an area with a high density of pig farms. *European Journal of Clinical Microbiology and Infectious Diseases*.

[81] van Loo I, Huijsdens X, Tiemersma E (2007). Emergence of methicillin-resistant *Staphylococcus aureus* of animal origin in humans. *Emerging Infectious Diseases*.

[82] Voss A, Loeffen F, Bakker J, Klaassen C, Wulf M (2005). Methicillin-resistant *Staphylococcus aureus* in Pig Farming. *Emerging Infectious Diseases*.

[83] Frana TS, Beahm AR, Hanson BM (2013). Isolation and characterization of methicillin-resistant *Staphylococcus aureus* from pork farms and visiting veterinary students. *PloS ONE*.

[84] Hanson BM, Dressler AE, Harper AL (2011). Prevalence of *Staphylococcus aureus* and methicillin-resistant *Staphylococcus aureus* (MRSA) on retail meat in Iowa. *Journal of Infection and Public Health*.

[85] O’Brien AM, Hanson BM, Farina SA (2012). MRSA in conventional and alternative retail pork products. *PLoS ONE*.

[86] Waters AE, Contente-Cuomo T, Buchhagen J (2011). Multidrug-resistant *Staphylococcus aureus* in US meat and poultry. *Clinical Infectious Diseases*.

[87] Pu S, Han F, Ge B (2009). Isolation and characterization of methicillin-resistant *Staphylococcus aureus* strains from louisiana retail meats. *Applied and Environmental Microbiology*.

[88] Bhargava K, Wang X, Donabedian S, Zervos M, da Rocha L, Zhang Y (2011). Methicillin-resistant *Staphylococcus aureus* in retail meat, Detroit, Michigan, USA. *Emerging Infectious Diseases*.

[89] Persoons D, van Hoorebeke S, Hermans K (2009). Methicillin-resistant *Staphylococcus aureus* in poultry. *Emerging Infectious Diseases*.

[90] Nemati M, Hermans K, Lipinska U (2008). Antimicrobial resistance of old and recent *Staphylococcus aureus* isolates from poultry: first detection of livestock-associated methicillin-resistant strain ST398. *Antimicrobial Agents and Chemotherapy*.

[91] Mulders MN, Haenen APJ, Geenen PL (2010). Prevalence of livestock-associated MRSA in broiler flocks and risk factors for slaughterhouse personnel in the Netherlands. *Epidemiology and Infection*.

[92] Feßler A, Scott C, Kadlec K, Ehricht R, Monecke S, Schwarz S (2010). Characterization of methicillin-resistant *Staphylococcus aureus* ST398 from cases of bovine mastitis. *Journal of Antimicrobial Chemotherapy*.

[93] Kwon NH, Park KT, Jung WK (2006). Characteristics of methicillin resistant *Staphylococcus aureus* isolated from chicken meat and hospitalized dogs in Korea and their epidemiological relatedness. *Veterinary Microbiology*.

[94] Kitai S, Shimizu A, Kawano J (2005). Characterization of methicillin-resistant *Staphylococcus aureus* isolated from retail raw chicken meat in Japan. *Journal of Veterinary Medical Science*.

[95] de Boer E, Zwartkruis-Nahuis JTM, Wit B (2009). Prevalence of methicillin-resistant *Staphylococcus aureus* in meat. *International Journal of Food Microbiology*.

[96] van Loo IHM, Diederen BMW, Savelkoul PHM (2007). Methicillin-resistant *Staphylococcus aureus* in meat products, the Netherlands. *Emerging Infectious Diseases*.

[97] Lozano C, López M, Gómez-Sanz E, Ruiz-Larrea F, Torres C, Zarazaga M (2009). Detection of methicillin-resistant *Staphylococcus aureus* ST398 in food samples of animal origin in Spain. *Journal of Antimicrobial Chemotherapy*.

[98] Kluytmans J, van Leeuwen W, Goessens W (1995). Food-initiated outbreak of methicillin-resistant *Staphylococcus aureus* analyzed by pheno- and genotyping. *Journal of Clinical Microbiology*.

[99] Bhat M, Dumortier C, Taylor BS (2009). *Staphylococcus aureus* ST398, New York City and Dominican Republic. *Emerging Infectious Diseases*.

[100] Huijsdens XW, van Dijke BJ, Spalburg E (2006). Community-acquired MRSA and pig-farming. *Annals of Clinical Microbiology and Antimicrobials*.

[101] Feßler AT, Kadlec K, Hassel M (2011). Characterization of methicillin-resistant *Staphylococcus aureus* isolates from food and food products of poultry origin in Germany. *Applied and Environmental Microbiology*.

[102] Osadebe LU, Hanson B, Smith TC, Heimer R (2013). Prevalence and characteristics of *Staphylococcus aureus* in connecticut swine and swine farmers. *Zoonoses and Public Health*.

[103] Bhalla A, Aron DC, Donskey CJ (2007). *Staphylococcus aureus* intestinal colonization is associated with increased frequency of *S. aureus* on skin of hospitalized patients. *BMC infectious diseases*.

[104] Weese JS, Avery BP, Reid-Smith RJ (2010). Detection and quantification of methicillin-resistant *Staphylococcus aureus* (MRSA) clones in retail meat products. *Letters in Applied Microbiology*.

[105] James SJ, Evans J, James C (2008). A review of the performance of domestic refrigerators. *Journal of Food Engineering*.

[106] Azevedo I, Regalo M, Mena C (2005). Incidence of Listeria spp. in domestic refrigerators in Portugal. *Food Control*.

[107] Lammerding AM (1997). An overview of microbial food safety risk assessment. *Journal of Food Protection*.

